# Supplementary material for: MetaRibo-Seq measures translation in microbiomes
Source: Nat Commun. 2020 Jun 29;11:3268. doi: 10.1038/s41467-020-17081-z (PMC7324362; doi:10.1038/s41467-020-17081-z)
Supplement: Supplementary file 10 — Supplementary Data 7 [file 41467_2020_17081_MOESM10_ESM.zip › File2/Confidence_High_Taxonomy/155941_out.krona.html]

Javascript must be enabled to view this page.

members
magnitude
magnitudeUnassigned
count
unassigned
taxon
rank

155941\_out

62

62
superkingdom
2

1239
phylum
62

186801
class
62

186802
62
order

family
1
216572

459786
genus
1

species
1
1945593

SRS077294\_contig\_number\_18706

family
61
541000

216851
61
genus

853

SRS012273\_contig\_number\_contig-100\_6705.222086SRS013098\_contig\_number\_27902SRS013940\_contig\_number\_15295SRS013965\_contig\_number\_169SRS014235\_contig\_number\_16365SRS014412\_contig\_number\_19470SRS014459\_contig\_number\_41246SRS014855\_contig\_number\_1399SRS015579\_contig\_number\_25565SRS016203\_contig\_number\_11780SRS016954\_contig\_number\_18522SRS017433\_contig\_number\_17460SRS019068\_contig\_number\_76068SRS019445\_contig\_number\_9446SRS020328\_contig\_number\_15312SRS021484\_contig\_number\_32122SRS022071\_contig\_number\_10592SRS024492\_contig\_number\_17490SRS042966\_contig\_number\_14436SRS045195\_contig\_number\_contig-100\_646.647SRS047014\_contig\_number\_16930SRS049959\_contig\_number\_53005SRS050752\_contig\_number\_contig-100\_4999.157293SRS051031\_contig\_number\_14739SRS054956\_contig\_number\_15528SRS056273\_contig\_number\_32649SRS058770\_contig\_number\_contig-100\_6998.126461SRS064557\_contig\_number\_12839SRS075878\_contig\_number\_20375SRS077194\_contig\_number\_4089SRS077502\_contig\_number\_3607SRS078242\_contig\_number\_22087SRS097920\_contig\_number\_24091SRS097958\_contig\_number\_10084SRS101376\_contig\_number\_24760SRS104084\_contig\_number\_11632SRS1041036\_contig\_number\_3282SRS1041090\_contig\_number\_2557SRS1041118\_contig\_number\_17383SRS1041136\_contig\_number\_9850SRS1055076\_contig\_number\_11052SRS140492\_contig\_number\_461SRS143148\_contig\_number\_5160SRS143523\_contig\_number\_25948SRS144362\_contig\_number\_13618SRS144537\_contig\_number\_34010SRS147022\_contig\_number\_14481SRS147346\_contig\_number\_59973SRS148253\_contig\_number\_8108SRS148784\_contig\_number\_23331SRS148874\_contig\_number\_3807SRS971276\_contig\_number\_19260
52
species

9
species

SRS011302\_contig\_number\_13211SRS018623\_contig\_number\_4426SRS063985\_contig\_number\_10881SRS075078\_contig\_number\_contig-100\_3073.190426SRS075984\_contig\_number\_contig-100\_2991.145833SRS098571\_contig\_number\_68775SRS098644\_contig\_number\_27494SRS1041037\_contig\_number\_16857SRS148159\_contig\_number\_43214
1971605
